# 3D-STED Super-Resolution Microscopy Reveals Distinct Nanoscale Organization of the Hematopoietic Cell-Specific Lyn Substrate-1 (HS1) in Normal and Leukemic B Cells

**DOI:** 10.3389/fcell.2021.655773

**Published:** 2021-06-30

**Authors:** Marta Sampietro, Moreno Zamai, Alfonsa Díaz Torres, Veronica Labrador Cantarero, Federica Barbaglio, Lydia Scarfò, Cristina Scielzo, Valeria R. Caiolfa

**Affiliations:** ^1^Malignant B Cells Biology and 3D Modeling Unit, Division of Experimental Oncology, IRCCS Ospedale San Raffaele, Milan, Italy; ^2^Nanomedicine Center NANOMIB, School of Medicine and Surgery, Università di Milano Bicocca, Milan, Italy; ^3^Unit of Microscopy and Dynamic Imaging, Centro Nacional de Investigaciones Cardiovasculares (CNIC), Madrid, Spain; ^4^B-Cell Neoplasia Unit and Strategic Research Program on CLL, Division of Experimental Oncology, IRCCS Ospedale San Raffaele, Milan, Italy; ^5^School of Medicine, Università Vita-Salute San Raffaele, Milan, Italy; ^6^Experimental Imaging Center, IRCCS Ospedale San Raffaele, Milan, Italy

**Keywords:** HS1, CLL, B cells, super-resolution, STED, phasor-FLIM, FRET

## Abstract

HS1, the hematopoietic homolog of cortactin, acts as a versatile actin-binding protein in leucocytes. After phosphorylation, it is involved in GTPase and integrin activation, and in BCR, TCR, and CXCR4 downstream signaling. In normal and leukemic B cells, HS1 is a central cytoskeletal interactor and its phosphorylation and expression are prognostic factors in chronic lymphocytic leukemia (CLL) patients. We here introduce for the first time a super-resolution imaging study based on single-cell 3D-STED microscopy optimized for revealing and comparing the nanoscale distribution of endogenous HS1 in healthy B and CLL primary cells. Our study reveals that the endogenous HS1 forms heterogeneous nanoclusters, similar to those of YFP-HS1 overexpressed in the leukemic MEC1 cell line. HS1 nanoclusters in healthy and leukemic B cells form bulky assemblies at the basal sides, suggesting the recruitment of HS1 for cell adhesion. This observation agrees with a phasor-FLIM-FRET and STED colocalization analyses of the endogenous MEC1-HS1, indicating an increased interaction with Vimentin at the cell adhesion sites. In CLL cells isolated from patients with poor prognosis, we observed a larger accumulation of HS1 at the basal region and a higher density of HS1 nanoclusters in the central regions of the cells if compared to good-prognosis CLL and healthy B cells, suggesting a different role for the protein in the cell types analyzed. Our 3D-STED approach lays the ground for revealing tiny differences of HS1 distribution, its functionally active forms, and colocalization with protein partners.

## Introduction

The hematopoietic cell-specific lyn substrate-1 (HS1) protein is the hematopoietic homologue of cortactin (Schnoor et al., 2018), belonging to the class II nucleation-promoting factor (NPF) family able to initiate branched actin network assembly by activating the Arp2/3 complex (Helgeson and Nolen, 2013).

The multi-domain sequence of HS1 is responsible for the recruitment of the protein in different mechanisms such as the activation of GTPases and integrins and the downstream signaling of BCR and TCR, and is indispensable for signaling events leading to actin assembly during immunological synapse (IS) formation (reviewed in Gomez et al., 2006; Castro-Ochoa et al., 2019). The HS1 sequence contains also a nuclear localization signal (van Rossum et al., 2005), for shuttling the transcription factor LEF-1 to the nucleus of myeloid cells (Skokowa et al., 2012). The N-terminal acidic region of HS1 mediates the connection of the Arp2/3 complex to actin intermediate filaments (IF), stabilizing newly formed branched actin networks (Uruno et al., 2003; Scherer et al., 2018). In response to various stimuli, a proline-rich domain interacts with SH2/SH3 domain-containing proteins, with phosphorylation of several tyrosine residues (Ruzzene et al., 1996).

HS1 functions are strictly regulated by posttranslational modifications. In B cells, HS1 is phosphorylated at residues Y378 and Y397 by BCR-activated Syk and Lyn kinases, followed by B-cell apoptosis (Yamanashi et al., 1997). Upon BCR-induced cross-linking and Syk activation, HS1 is localized in membrane lipid rafts, suggesting an adapter role in the recruitment and assembly of actin (Hao et al., 2004). In T cells, phosphorylation links HS1 to multiple signaling proteins, including Lck, PLCγ1, and Vav1, and is essential for the stable recruitment of Vav1 to the IS (Gomez et al., 2006).

Chronic lymphocytic leukemia (CLL) is the most common leukemia in the Western world and affects mainly elderly patients (Tausch et al., 2014). Most patients with CLL are diagnosed with this disease at the early stage and managed with active surveillance; however, the individual course of patients is very heterogeneous, and their probability of needing treatment is difficult to be anticipated at diagnosis. For this reason, different prognostic factors have been explored over time (Chen and Puvvada, 2016). Recently, the immunoglobulin heavy variable gene (IGHV) unmutated status has been defined as the biomarker with the strongest effect on time to first treatment prognostication (Condoluci et al., 2020).

CLL cells are clonal CD5^+^ B lymphocytes that accumulate, progressively expand, and traffic between peripheral blood, bone marrow, and secondary lymphoid organs (Burger and Gribben, 2014; Caligaris-Cappio et al., 2014), where they proliferate by sensing and reacting to the microenvironment (i.e., stromal, endothelial, and immune cells), likely undergoing a major cytoskeleton rearrangement (Calissano et al., 2009; Herishanu et al., 2011; Ponzoni et al., 2011). The continuous trafficking of CLL cells from tissue back to circulation induces a phenotype that possibly contributes to disease progression and chemo-resistance (Burger, 2010; Herishanu et al., 2011; Ponzoni et al., 2011).

The implication of the cytoskeleton in the dynamic behavior of CLL cells was first observed in early studies showing that CLL cells manifest anomalous motility, cap formation (Stark et al., 1984), and aberrant cytoskeleton rearrangement (Caligaris-Cappio et al., 1986).

These mechanisms are controlled by cytoskeleton regulatory molecules, as HS1, which we reported to be able to interact with cytoskeleton adapters such as Vimentin and HIP-55 in normal and leukemic B cells (Muzio et al., 2007). Moreover, HS1 was identified as a prognostic marker in CLL (Scielzo et al., 2005) depending on its activation status; active/inactive forms correlate with favorable or adverse prognosis, respectively (Yamanashi et al., 1993, 1997; Scielzo et al., 2010).

We demonstrated that downregulation of HS1 expression interferes with CLL cell secondary lymphoid organ infiltration and leads to increased bone marrow homing, which is associated with impaired cytoskeletal activity (Scielzo et al., 2010; ten Hacken et al., 2013). Further underlining the potential clinical significance of HS1, more recently, it has been also shown that its association with ROR1 enhances CLL cell migration (Hasan et al., 2017).

These mechanisms imply an intracellular dynamics and redistribution of HS1 and its active/inactive forms that have not been studied in detail to date in B cells. Confocal microscopy studies have given only an overall visualization of HS1 in the extranuclear space. However, up to now little is known about the nanostructural features of HS1 in primary CLL cells obtained from patients with diverse prognoses. It is also missing a direct comparison with the HS1 patterns in model cell lines that, in contrast to primary CLL cells, are prone to genetic manipulation and, therefore, allow studies on the dynamics of HS1 relocations and interactions. The prerequisite for approaching this level of investigation is to determine how and where the total endogenous HS1 distributes in primary CLL cells and whether we find differences between normal B cells and CLL cells originated from patients with diverse prognoses. As a second step, we also aim at comparing the HS1 nanostructure organization in leukemic MEC1 cells, since this cell line can be used for controlling the expression of specific active/inactive HS1 forms and follow their intracellular dynamics.

In this work, we show for the first time that a 3D-STED super-resolution approach is instrumental for revealing HS1 clustering in primary CLL and B cells and a concentration gradient of the protein toward the adhesion sites, features that are well recapitulated in the MEC1 CLL cell line.

## Materials and Methods

### Cells and Human Primary Sample Purification

CD19 cells were negatively selected from fresh pheripheral blood from patients or healthy donors using the RosetteSep B-lymphocyte enrichment kit (StemCell Technologies, Vancouver, Canada). Human healthy cells were further negatively selected using B-lymphocyte enrichment kit (StemCell Technologies, Canada). The purity of all preparations was always higher than 99%, and the cells co-expressing CD19 and CD5 on their surface as assayed by flow cytometry (Navios Beckman Coulter Life Science, Indianapolis IL, United States) preparations were virtually devoid of natural killer cells, T lymphocytes, and monocytes.

The MEC1 cell line was obtained from Deutsche Sammlung von Mikroorganismen und Zellkulturen GmbH (DSMZ) and cultured in RPMI 1640 medium (Euroclone, Milan, Italy) supplemented with 10% volume/volume (v/v) fetal bovine serum (FBS) and 15 mg/ml gentamicin (complete RPMI) at 37°C and 5% CO_2_. MEC1-HS1-YFP was generated by transfecting MEC1 cells, taking advantage of Nucleofector Technology (AMAXA) with the use of the program X-001, solution V.

The Vivid Colors^TM^ pcDNA^TM^6.2/N-YFP-DEST Vector expressing the HS1 gene (Thermo Fisher Scientific, Waltham, MA, United States) was used for transfection and blasticidin (Thermo Fisher Scientific, United States) for the antibiotic selection.

### Immunofluorescence Staining

Primary cells and cell lines (3 × 10^6^) were seeded in medium on polyornithine:PBS (1:5)-coated coverslips (22 × 22-mm high-precision glass, code: 0101050, Marienfeld, Germany) and incubated for 2 h at 37°C and 5% CO_2_. For single immunostaining of endogenous HS1, after removing the incubation medium, cells were washed with PBS, fixed with PFA 4%, incubated 15 min in the dark, and permeabilized in blocking buffer (blocking buffer: 0.1% w/v BSA, 10% v/v FBS in PBS), containing 0.3% v/v Triton-X 100 (Sigma-Aldrich, Merck, Germany), to limit unspecific antibody binding. Samples were incubated overnight at 4°C with a primary monoclonal anti-mouse HS1 Ab:PBS solution (code: 610541, BD Biosciences, San Jose, CA, United States) then labeled with an anti-mouse-Alexa 488 (code: A11001, Invitrogen, Thermo Fisher Scientific, United States) or anti-mouse-Alexa 568 (code: A11004, Invitrogen, Thermo Fisher Scientific, Waltham, MA, United States) for 2 h at RT and in the dark. The lack of cross-reactivity for cortactin was checked by applying the immunostaining protocol to fibroblasts, which have a high expression of cortactin but lack HS1 (Supplementary Figure 1).

For double HS1-Vimentin immunolabeling in MEC1-YFP-HS1 cells, we followed the above protocol modifying the fixation step to PFA 1% for 15 min. Samples were incubated overnight at 4°C with primary anti-goat YFP (600-101-215M, Thermo Fisher Scientific, United States) and monoclonal anti-mouse Vimentin (code: SC 6260, Santa Cruz Biotechnology, Santa Cruz, CA, United States), followed by incubation for 2 h at RT, in the dark with anti-goat-Alexa 568 (code: A11057, Invitrogen, Thermo Fisher Scientific, United States) and anti-mouse-Alexa 532 (code: A11002, Invitrogen, Thermo Fisher Scientific, United States).

For FRET experiments, MEC1 cells (4 × 10^6^) were co-immunostained for endogenous HS1 and Vimentin. Fixation was in PFA 1% for 30 min at RT. Samples were blocked and incubated as above with a primary monoclonal anti-Vimentin-mouse (Santa Cruz Biotechnology, Inc., Santa Cruz, CA, United States) and anti-HS1-goat Abs, then labeled with anti-mouse-Alexa 546 and anti-goat-Alexa 488.

For microscopy, we used ProLong Gold or Diamond antifade reagents (Invitrogen, Thermo Fisher Scientific, United States) for B and CLL or Diamond for MEC1-HS1-YFP cells as mounting media.

### Super-Resolution 3D-STED Microscopy

We used a gated STED-3X-WLL SP8 microscope (Leica Microsystems, Wetzlar, Germany) and a HC Pl Apo CS2 100x/1.40 oil objective for all experiments. The microscope was equipped with 592- and 660-nm depletion lasers, and the excitation was provided by a pulsed white laser. The acquisition software was LAS X 3.5.6.21594.

For cluster analysis on Alexa 488 immunolabeled HS1 in CLL and B cells, X,Y,Z depletion was obtained by a STED 592-nm laser set at 100% output in X, Y, and Z. Excitation at 489 nm was performed by a white laser fixed at 3%, applying a gated unidirectional resonant scanning mode with a 16-line average and 8-frame accumulation at 8,000 Hz scan speed. Fluorescence (508–555 nm) was collected using a HyD spectral detector in standard mode and a gating of 0.1. A zoom 2 was applied to acquire Z-stacks of 13 optical sections of 968 × 968 pixels at the center of the cells with a voxel size of 60 × 60 × 100 nm. This setting was chosen among others to maximize axial resolution and minimize photobleaching, autofluorescence, and reflections, keeping the white laser excitation at the minimum yet being capable of maintaining the same settings for all cells, which showed variable intensities (i.e., amount of stained HS1). Depletion laser power, image format, zoom, and scanning conditions were optimized according to the same principle to obtain minimal photobleaching and Z-distortion and define conditions applicable to all samples for a total of 220 cells. The image resolution under the conditions optimized for our samples was determined by measuring the X,Y,Z PSF in a 23-nm nanobead sample coated with the Alexa 488 fluorophore (GATTAquant GmbH, Germany) (Supplementary Figure 2).

To reconstruct the total intracellular distribution of Alexa 488 immunolabeled HS1 in primary CLL and B cell and in YFP-HS1 MEC1 samples, we acquired Z-stacks of about 4–7 μm under the above conditions. For Alexa 568-endogenous HS1 MEC1 cells, we depleted the samples at 660 nm. Line and frame accumulation was adapted to the Z-stack depth to minimize photobleaching by testing forward and backward scanning (Supplementary Movie 1), and voxel size was optimized for deconvolution.

For 2D-STED HS1-Vimentin colocalization in MEC1 cells, we worked with the 660-nm depletion line between frames, at 700 Hz unidirectional speed. A summary of the super-resolution acquisition conditions is given in Supplementary Table 1.

### Image Analysis

For cluster analysis, undeconvolved STED Z-stacks were divided in single-cell ROIs. Each ROI was analyzed individually and, when necessary, postprocessed for bleaching by exponential fit Fiji (ImageJ) plugin. ROI Z-stacks with ≥10% bleaching were discarded. Cluster size was determined by IMARIS software (9.1.2 Oxford Instruments plc, Oxon OX13 5QX, United Kingdom). The lowest detectable limit was set equal to four voxels, based on the X,Y,Z resolution obtained for the 23-nm Alexa 488 beads (Supplementary Figure 2). The total volume of each Z-stack was determined by local intensity thresholding above the background.

Deconvolution was performed on single-labeled whole-cell STED Z-stacks of primary CLL, B (Alexa 488-endogenous HS1) and MEC1 (Alexa 568-endogenous HS1 or YFP-HS1) cells and on the 2D-STED images of double immunolabeled MEC1 cells (Alexa 568-endogenous HS1 + Alexa 532-endogenous Vimentin). For deconvolution, we used the Huygens software (Scientific Volume Imaging BV, Hilversum, The Netherlands), applying the GMLE algorithm to the STED Z-stacks and the CLME algorithm to the 2D-STED images.

Colocalization analysis was performed by the ImageJ-Fiji Coloc 2 plugin software, and statistical analysis by GraphPad Prism software (GraphPad Software, San Diego, CA, United States).

### FRET by Two Photon Phasor-FLIM

The two-photon laser scanning fluorescence microscope for FLIM experiments was assembled at the Laboratory for Fluorescence Dynamics (Irvine University, CA, United States) and was described in detail previously (Caiolfa et al., 2007; Digman et al., 2008). A 40 × 1.2 NA oil-immersion objective (Carl Zeiss, Inc., Germany) was used to acquire fluorescence and lifetime decays at 905-nm excitation. Before measurement, a slide with concentrated fluorescein, pH 9.0, was measured as the standard lifetime and compared with that of 4.04 ns, determined separately in a photoncounting spectrofluorometer (PC1; ISS Inc., Champaign, IL, United States). Images were collected with 0.75 and 1.84 mW laser power at the sample, in 256 × 256 pixel format, equivalent to 32 × 32 μm^2^, with a pixel residence time of 16 or 64 μs, depending on the experiment. The total frame acquisition time was 1–4 s to avoid photobleaching. Several frames (10–30) were acquired and averaged for the analysis by SimFCS software (Laboratory for Fluorescence Dynamics Irvine University, CA, United States). FLIM analysis for deriving the FRETeff was performed using the phasor method (Caiolfa et al., 2007; Digman et al., 2008). Statistical analysis was performed by GraphPad Prism software (GraphPad Software, San Diego, CA, United States).

## Results

### Immunolabeled Endogenous HS1 Is Recognized in Clusters in Primary CLL and Normal B Cells

We isolated primary leukemic B cells from PB samples obtained from patients (*n* = 11) (Supplementary Table 2; Hallek et al., 2008) with CLL and from healthy donors (*n* = 6) for determining the endogenous HS1 localization at the increased resolution of the 3D-STED imaging. To evaluate possible differences in HS1 activity linked to its localization and intracellular nanostructure in CLL, we stratified the patients based on the mutational status of the IGHV being HS1 downstream the B cell receptor (Castro-Ochoa et al., 2019). We considered that, in CLL patients managed with active surveillance, the IGHV unmutated status is the biomarker with the strongest effect on time to first treatment prognostication (Condoluci et al., 2020). Accordingly, mutated IGHV was associated with patients with good prognosis (mCLL, *n* = 6), and unmutated IGHV identified patients with poor prognosis (uCLL, *n* = 5).

**FIGURE 1:**
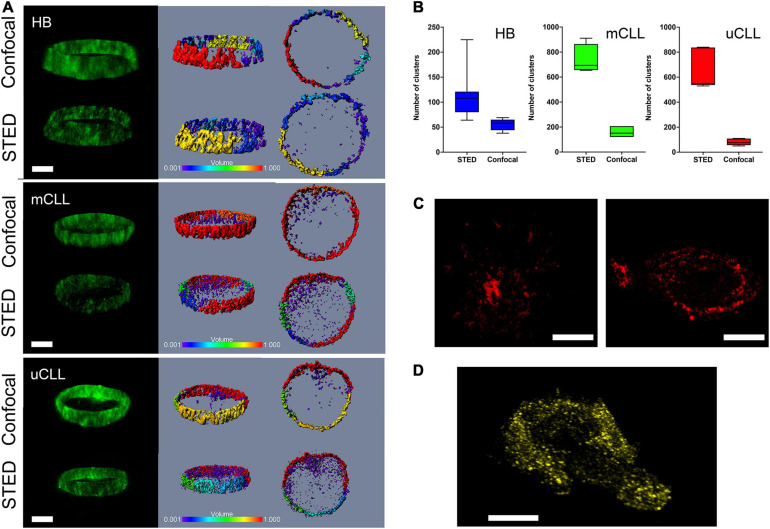
Immunolabeled endogenous HS1 is recognized in clusters in primary CLL and normal B cells. **(A)** Comparison of confocal and STED Z-stacks acquired in central cell regions of Alexa 488-endogenous HS1 in HB, mCLL, and uCLL cells. Fluorescence intensity X,Z stereo view (green pseudo color, left), cluster size stereo view X,Z and X,Y (rainbow pseudo color in μm^3^, middle and right). Scale bar 4 μm. **(B)** Histogram of cluster number determined by the IMARIS software in the three confocal and STED examples shown in **(A)**. **(C)** HS1 clusters detected by STED in two representative MEC1 cells stained for Alexa 568-endogenous HS1. Scale bar 4 μm. **(D)** STED Z-stack stereo view of a representative MEC1 cell overexpressing YFP-HS1. Scale bar 4 μm.

Endogenous immunolabeled-Alexa 488 HS1 in B and CLL cells was compared by confocal and STED microscopy (Figure 1A), examining sagittal sections of 1.3 μm. Confocal images depict a rough distribution, heavily distorted along the *Z*-axis of the protein that accumulates in the extranuclear volume. In these images, however, we can detect disperse clusters penetrating in the nucleus with an apparent volume of about 0.001 μm^3^, suggesting that the protein would have a cluster-like distribution all over the cell. From this initial observation, we concluded that the information attainable by conventional 2D-STED, although at the highest X,Y resolution, is biased by the lack of resolution along the *Z*-axis, merging the signal spread perpendicularly inside the cell. Moreover, considering that primary CLL cells are roundish and small (about 5–7 micron), with the nucleus that occupies most of the intracellular space, and that we expect inter- and intra-patient heterogeneity, a single X,Y 2D-STED plane would give insufficient information to derive conclusions about the distribution of the HS1 protein from a single patient, and from different patients. Therefore, we accepted the compromise of reducing X,Y resolution and introduced depletion along the *Z*-axis, obtaining a more isotropic 3D-STED voxel (Supplementary Figure 2) that moderates the imaging distortions, allows to collect optical sections, and increases the significance of our analysis. Figure 1A shows the related 3D-STED images in which the HS1 clustering is evident throughout the cell section. The discrete distribution of HS1 in the example in Figure 1A was quantified by the number of identified clusters per imaged volume in 3D-STED Z-stacks in Figure 1B.

We compared the results from primary cells, with the distribution of the immunolabeled endogenous HS1 in MEC1 cells, observing a similar pattern of discrete clusters (Figure 1C). Additionally, to exclude that HS1 clustering was, to some extent, induced by the immunostaining protocol, we also investigated the distribution of a YFP-labeled HS1 overexpressed in MEC1 cells. The genetically labeled protein was found again in clusters, recapitulating the nanostructural features observed in primary CLL cells (Figure 1D).

On the one hand, these results provide the first high-resolution visualization of endogenous HS1 recruited in distinct clusters in primary normal and leukemic B cells. On the other hand, they also suggest that MEC1 cells might be a suitable model for characterizing the role of the active protein by means of genetically modified lines.

### Endogenous HS1 Clusters Differentiate uCLL From mCLL and Normal B Cells

We pursued an analysis of single cells in single-patient samples, applying a 3D-STED protocol optimized as above to characterize HS1 clusters in B, uCLL, and mCLL cells and assess in-sample and interpatient heterogeneity. Primary cells were plated on precoated polyornithine coverslips and Alexa 488-immunostained for HS1. Firstly, each sample was inspected by tiled confocal microscopy to estimate the number of cells in the sample, cellular integrity, density, and dispersion on the coverslip (Supplementary Figure 3). We detected a minor number of cell contacts that were easily distinguishable from rare immune-synapse-like events, possibly due to minor T cell contamination (Supplementary Figure 3). In both cases, cells engaged in pairs were excluded from the analysis.

**FIGURE 2:**
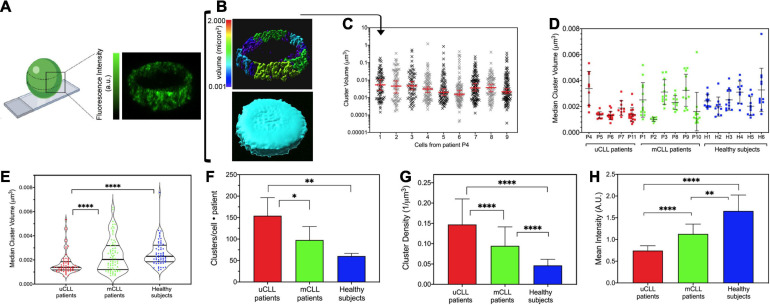
Cluster analysis in primary CLL and healthy B cells. **(A)** Schematic representation created by www.biorender.com that illustrates the position chosen for the analysis of Alexa 488-immunolabeled HS1 clusters (right) and a representative single-cell STED Z-stack (left). **(B)** Example of the IMARIS cluster analysis (rainbow volume scale, top) and of the total volume enclosing the clusters used for data normalization (turquoise, bottom). Size is expressed in μm^3^. **(C)** Representative clusters measured in nine primary cells from patient P4. Data are plotted in log-Y scale, and the median and interquartile ranges are shown (red bars). **(D)** Scatter plot illustrating the median cluster size resulting from the analysis of the cells of each patient. Each dot is the median cluster size in a single cell. Black bars indicate the mean (±SD) value for each patient sample. Patients with poor prognosis, uCLL: P4, P5, P6, P7, P11; patients with good outcome, mCLL: P1, P2, P3, P8, P9, P10; normal subjects, HB1–HB6. **(E)** Violin plot resuming the cluster size distribution per group of patients and healthy subjects. Each dot is the median cluster size in a single cell and patient. Black lines: median distribution with quartiles. uCLL vs. HB: ****P < 0.0001; uCLL vs. mCLL: ****P < 0.0007; mCLL vs. Healthy: not significant. **(F)** Histogram of total number of clusters normalized by the number of cells and number of subjects included in each group (mean ± SD). uCLL vs. Healthy: **P < 0.0012; uCLL vs. mCLL: *P < 0.0193; mCLL vs. Healthy: not significant. **(G)** Histogram of cluster densities (mean ± SD) per group of subjects. Data are normalized for the volume of the Z-stack of each cell, for the number of cells, and for the number of patients/subjects in each group. uCLL vs. Healthy: ****P < 0.0001; uCLL vs. mCLL: ****P < 0.0001; mCLL vs. Healthy: *P* < 0.0001. **(H)** Histogram of the fluorescence intensities (mean ± SD) Data are normalized for the number of cells and number of patients/subjects in each group. uCLL vs. Healthy: ****P < 0.0001; uCLL vs. mCLL: ****P < 0.0013; mCLL vs. Healthy: **P < 0.0001. See Supplementary Table 1 for patients’ details. Two-way ANOVA was applied for the statistical significance in **(E–H)**. uCLL 64 cells from 5 patients; mCLL 60 cells from 6 patients; Healthy 66 cells from 6 normal subjects.

For cluster analysis, we standardized data collection by acquiring a fixed volume above and below the center of each single cell (Figure 2A) and used the Imaris software for calculating the cluster number, size (Figure 2B, top), and volume of the imaged cellular section (Figure 2B, bottom).

In all cells, cluster size followed a non-parametric distribution which is well represented by a median value (Supplementary Figure 4). Figure 2C illustrates these results from the analysis of nine cells from patient P4 in a log-Y scatter plot (Supplementary Table 2). In these cells, cluster size varied from a minimum of 0.0002 μm^3^ to a maximum of 1 μm^3^, giving median values ranging from 0.0015 to 0.0053 μm^3^ (red bars in Figure 2C). The analysis was repeated on all single cells in patient samples, and the median values were compared (Figure 2D). In the group of patients P4, P5, P6, P7, and P11 associated with poor CLL prognosis (Supplementary Table 2), HS1 clusters were small and less variable in size, except for patient P4. Despite the unmuted IGHV identity, cells from patient P4 showed a large variability of HS1 clusters, which was not observed in patient P6 that had the same IGHV identity (Figure 2D and Supplementary Table 2). This discrepancy is likely due to our approach of identifying the patients’ groups without considering other prognostic factors (Chen and Puvvada, 2016). Nevertheless, in the primary CLL cells from this group of patients, we found significantly smaller HS1 clusters (Figure 2E), which were clearly distinguishable from the HS1 clusters identified in mCLL patients with good prognosis and B cells from healthy donors. HS1 cluster size in the latter two groups, mCLL cells (patients P1, P2, P3, P8, P9, P10) and normal B cells (healthy H1, H2, H3, H4, H5, H6), was largely scattered (Figure 2D) and did not allow to distinguish the two groups (Figure 2E).

In addition to the size, we also counted the number of HS1 clusters in single-cell sections and found that HS1 forms not only smaller but also more numerous clusters in uCLL cells as compared to mCLL and normal B cells (Figure 2F). Because of these differences, the density per volume unit of the HS1 protein is higher in uCLL cells (Figure 2G).

Altogether, the results indicate that endogenous HS1 has a distinct distribution in uCLL derived from patients with poor prognosis. Only in these cells, the protein is packed in a high number of very small clusters forming a dense, punctuate filling of the extranuclear regions. Interestingly, the fluorescence intensity of the Alexa488-HS1 clusters in uCLL is much lower than in mCLL and normal B cells, suggesting that overall the concentration of the protein in the analyzed regions is lower (Figure 2H).

With this analysis, we can also point out to some differences between mCLL and normal B cells. Although at the level of the HS1 cluster size these two groups are not clearly different, the number of clusters (Figure 2F) and the density of the protein in the central cell regions (Figure 2G) are higher in mCLL cells than in normal B cells. Finally, also in mCLL cells, the intensity of the immunolabeled HS1 is lower than in normal B cells, indicating again a different organization of the protein among the three groups (Figure 2H).

### HS1 Clusters Form a Concentration Gradient Toward the Adhesion Sites

The differences of HS1 clustering observed in central regions of primary uCLL, mCLL, and normal B cells might be indicative of differences in the total intracellular distribution of the protein among the three groups of subjects. Thus, based on previous evidences on the role of HS1 in cell adhesion and its implication in cytoskeleton rearrangements in CLL cells (Scielzo et al., 2010), we asked whether the HS1 distribution would change toward the adhesion sites of normal B and CLL cells.

**FIGURE 3:**
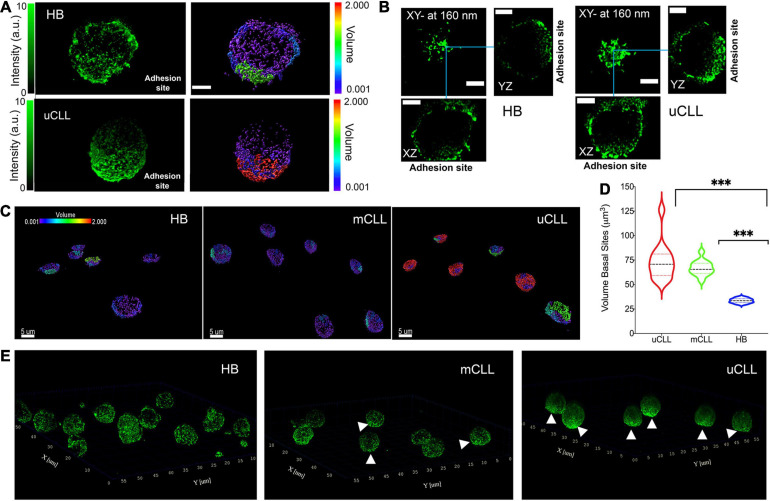
Concentration gradient of HS1 clusters toward the adhesion sites. **(A)** Representative 3D rendering of STED Z-stacks acquired on a single HB and uCLL cells. The fluorescence intensity (green pseudo color) is compared with the cluster size reconstruction by IMARIS software (rainbow pseudo color in μm^3^). Scale bar 2 μm. **(B)** Orthogonal projections of the cells shown in **(A)** from 0 to 160 nm above the basal side. Blue lines indicate the orthogonal cross. Scale bar 2 μm. **(C)** Representative 3D rendering of STED Z-stacks acquired at the lowest planes above the coverslip and over a field of view of six or seven cells in parallel experiments. HS1 accumulates in unresolved assemblies in cells obtained from a patient with poor prognosis (uCLL) compared to the more homogeneous cluster distribution of the protein in healthy B cells (HB) and in CLL cells from a patient with good outcome (mCLL). The volume pseudo rainbow scale is in μm^3^. **(D)** Violin plot distributions of the cluster size at basal side. HB 7 cells, median 33.5, interquartile 31.6/35.6; mCLL cells 13 from two patients, median 65.5, interquartile 61.4/71.8; uCLL cells 13 from two patients, median 77.8, interquartile 59.3/81.2. Two way ANOVA ^∗∗∗^*P* = 0.0003. **(E)** Whole-cell 3D-rendering of STED Z-stacks showing the distribution of endogenous HS1 in healthy B (HB) and CLL cells obtained from patients with good (mCLL) or poor (uCLL) prognosis. The white arrows point to the HS1 accumulation.

We examined regions in contact with the cell support and observed an accumulation of HS1 clusters in healthy B cells (Figure 3A, top, Figure 3B, left). In uCLL cells, HS1 accumulate more toward the adhesion site, coalescing in large assemblies (Figure 3A, bottom, Figure 3B, right). According to the volume analysis (Figure 3C), the accumulation of the protein at the adhesion sites is much higher in CLL cells in comparison to healthy B cells (Figure 3D).

The whole-cell 3D rendering of STED Z-stacks gives an overall view of the HS1 cluster distribution (Figure 3E and Supplementary Movies 2–4). It is noteworthy that the cellular volume delineated by the distribution of immunolabeled HS1 is comparable to that obtained in SEM (Supplementary Figure 5), demonstrating how the 3D-STED protocol developed in this work minimizes the volumetric distortions typical of fluorescence microscopy, which have detrimental effects on the analysis of protein clusters and small aggregates.

The 3D-STED images show, at unprecedented resolution, how the endogenous protein clusters fill the tiny volume surrounding the nucleus. In uCLL cells (Figure 3E, right), HS1 clusters become an unresolved and dense surface toward the basal side, adhering to the coverslip. A similar concentration gradient is not observed in healthy B cells (Figure 3E, left) and in mCLL cells (Figure 3E, middle).

These results agree with the previous observations suggesting the recruitment of HS1 for cell adhesion, and its co-actor role in the cytoskeleton rearrangement. Moreover, the results also highlight tiny differences at the level of the total endogenous HS1 distribution, distinguishing a clear concentration gradient in uCLL cells from patients with unfavorable prognosis.

### MEC1 Cell Line Is a Valid Model to Recapitulate the Activity of Endogenous HS1 in Primary CLL Cells

**FIGURE 4:**
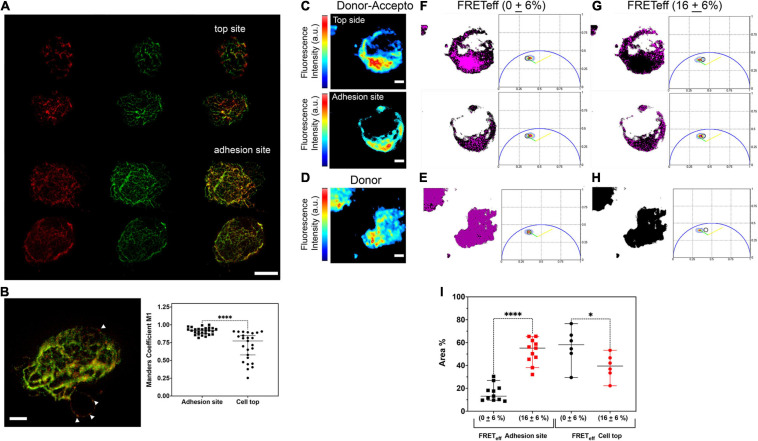
MEC1 cells recapitulate the HS1 gradient of primary CLL cells. **(A)** Immunofluorescence 2D-STED images taken at the top and adhesion sites of representative MEC1 cells showing a colocalization gradient of endogenous HS1 and Vimentin. AntiHS1Ab568 (red), antiVimentinAb532 (green), and merged panels. Scale bar 4 μm. **(B)** Representative 3D rendering of a Z-stack of a double HS1-Vimentin-immunostained MEC1 whole cell (left, white arrows point to the HS1 accumulation), and plot of Manders’ coefficients M1, proportional to the amount of fluorescence of the red channel (HS1) co-localizing with the green channel (Vimentin) in cell sections taken at the adhesion and top sites (right). Adhesion site 27 cells, top site 30 cells. Bars: mean ± SD. Mann–Whitney test *P* < 0.0001. Scale bar 2 μm. **(C–H)** Representative two-photon phasor-FLIM-FRET analysis of immunostained endogenous HS1-Alexa488 (donor) and endogenous Vimentin-Alexa 546 (acceptor) in an MEC1 cell. Scale bars 4 μm. **(I)** Scatter plot of the normalized cell areas with FRETeff either negligible (0 ± 6%) or equal to 16 ± 6% measured at the adhesion and top sites in single cells. Areas are reported as % ratio between number of pixels with FRET (pink mask as in panels **E–H**) over the total image area (black mask as in panels **E–H**) at the adhesion and top sites. Bars: mean ± SD. ^****^P < 0.0001; ^∗^*P* = 0.0466. Adhesion site (0 ± 6%) and (16 ± 6%): 11 cells; Top site (0 ± 6%) and (16 ± 6%): 6 cells.

With HS1 being involved in cytoskeleton dynamics, there is the need of valid cellular models expressing fluorescent HS1 variants, which cannot be obtained in primary cells. With this aim, we validated the MEC1 CLL line as a suitable model for live cell studies. Our results demonstrate that the tiny cluster distribution of endogenous HS1 observed in primary cells is maintained in immunolabeled MEC1, and it is similar to the distribution of YFP-HS1 overexpressed in these cells (Figure 1). We have also shown a concentration gradient of HS1 in primary cells toward the adhesion sites, supporting the role of HS1 as a cytoskeleton adapter. Therefore, we investigated the distribution of endogenous HS1 in comparison with Vimentin by co-immunolabeling MEC1 cells (Figure 4). 2D-STED images were acquired at the top plane (Figure 4A, top) and nearby the coverslip (Figure 4A, bottom). Our results suggest higher HS1-Vimentin colocalization toward the adhesion sites. Colocalization was also evident in the whole-cell stack (Figure 4B, left), and it was replicated in a high number of cells (Figure 4B, right).

We have also asked whether the colocalization of HS1 and Vimentin reports on a direct interaction between the two proteins, as previously suggested (Muzio et al., 2007). For this purpose, we used the two-photon phasor FLIM-FRET approach (representative experiment in Figures 4C–H and Supplementary Figure 6). Cells were immunolabeled for HS1 (FRET donor Alexa488) and Vimentin (FRET acceptor Alexa 546), and the fluorescence intensity image of a double-stained cell was recorded at the adhesion and top sites (Figure 4C). The fluorescence intensity image of a cell stained only for the unquenched HS1-Alexa 488 donor was acquired as control (Figure 4D). In parallel, the fluorescence lifetime decays were recorded and the entire distributions of the donor fluorescence lifetime in double (Figures 4F,G) or single-stained (control Figures 4E,H) cells were reported by a contour plot in polar coordinates. In these plots, the green lines represent the calibration obtained by measuring the lifetime distributions of donor-only cells at decreasing staining intensities in the presence of autofluorescence (unstained cells). The calibration measures the decrease of the donor lifetime due to photobleaching. When donor quenching is due to FRET, the phasor distribution moves outside the green line, and the yellow lines in the phasor plots mark the position expected for 50% FRET efficiency in the presence of variable donor intensities as previously illustrated (Caiolfa et al., 2007).

In each phasor plot, the total fluorescence lifetime distribution is explored by a round cursor. The size of the round cursor was set according to the dispersion of the lifetime in the control cells which, in our experiments, was 6% (Figure 4E). The pixels selected inside the round cursor were then localized in the associated images by pink masks. As represented in this example of a double-stained cell (Figures 4F,G), pixels that fall into the uncertainty area (control pure donor, Figure 4E) were considered to give a negligible value of FRETeff = 0 + 6%. In contrast, pixels outside the area of the control (Figure 4H) gave an average FRETeff = 16 + 6%. While at the top of the cells FRETeff was mostly negligible, large areas occupied by interacting HS1 and Vimentin were again detected at the cell adhesion sites, indicating an interaction gradient that support the recruitment of HS1 in the cytoskeleton reorganization for cell adhesion (Figure 4I).

Thus, the results in MEC1 cells parallel the unique observations in primary CLL cells and validate this CLL line as a suitable tool for dissecting the specific role of the active/inactive HS1 forms.

## Discussion

CLL, the most common adult leukemia in the Western countries, is clinically very heterogeneous and is still uncured. It may express a pre-leukemic form (Caligaris-Cappio and Ghia, 2008; Dagklis et al., 2009; Rawstron, 2009) or appear with an indolent clinical course or as a progressive disease that in some cases transforms into an aggressive high-grade lymphoma (Dagklis et al., 2009; Rawstron, 2009; Fazi et al., 2011). CLL clinical course reflects biological heterogeneity, and patients are usually classified in two main subsets depending on good or poor prognosis, which are based on a set of prognostic factors for the disease outcome and survival. Several studies have demonstrated that differences in the clinical course of the disease can be partially explained by the presence (activation) or absence (inactivation) of some biological markers with a prognostic value, including HS1 (Damle et al., 1999; Lanham et al., 2003; Wiestner et al., 2003; Apollonio et al., 2014).

HS1 has emerged as a key molecule involved in B-cell migration and specific organ homing especially to the bone marrow. HS1 acts as an actin regulator of the immune synapse in T cells (Gomez et al., 2006) and regulates adhesion, lytic synapse formation, cytolytic activity, and chemotaxis in NK cells (Butler et al., 2008). In both normal and leukemic B cells, HS1 interacts with cytoskeleton adapter proteins involved in cytoskeleton reorganization (Muzio et al., 2007). By dissecting HS1 molecular function in CLL cells, it was established that HS1 takes part in the formation of a complex together with the ZAP-70 kinase and several cytoskeleton adapters (HIP-55 and cortactin) and that it co-localizes with F-actin and Vimentin, thereby showing an enrolment in regulation of the B-cell cytoskeleton (Muzio et al., 2007). While a low Vimentin content of CLL cells correlates with an increased survival, the phosphorylation levels of HS1 relate to the clinical course of patients with CLL, with the hyperphosphorylated form of HS1 being associated with a more aggressive disease (Scielzo et al., 2005). Yet, it is still unclear to what extent HS1 has different functions in CLL and healthy B cells and what determines its prognostic value. Moreover, unraveling HS1 expression and activation could have potential implications also in other leukaemias and lymphomas in particular for T cell leukaemias, and for novel immune/cell therapies, such as CAR-T (El Hajj et al., 2020) and cell therapy (Cerrano et al., 2020), all these aspects have not been explored so far.

Thus, it is important to develop state-of-the-art approaches for complementing the study of HS1 as a structural adapter and signaling molecule related to immune response, migration, adhesion, transendothelial migration, and antigen recognition. With this objective in mind, we obtained a high-resolution visualization of HS1 in primary normal and leukemic cells, and then we compared the results with the distribution of the protein in the MEC1 cell line, also by overexpressing the YFP-HS1 fluorescent variant. Our goal has been twofold: to provide the first refined structural pattern of the protein in primary cells and to investigate whether the MEC1 leukemic cell line can be considered a suitable model in which it is possible to dissect the functions of the specific phosphorylated forms of HS1 by genetically modified expression. Moreover, we divided CLL cells according to the IGHV mutational status of the donor patients in mCLL and uCLL and established a quantitative protocol that has allowed to detect significant differences in the distribution and organization of endogenous HS1 among cells from the two groups of patients and in comparison with normal B cells.

Our results reveal with an unprecedented level of details that endogenous HS1 in primary CLL cells forms nanoclusters all over the extranuclear cell body, with a density gradient toward the adhesion site of the cells. We were able to correlate the HS1 clustering with the mutational state of IGHV and show that in uCLL cells from patients with adverse outcome HS1 clusters are distinguishably smaller, yet highly packed and recruited at the cell adhesion plane. In contrast, in mCLL, cells from patients manifesting a mild prognosis, HS1 clustering in terms of size, number, and concentration gradient showed intermediate values between the extreme features of uCLL cells and the normal features of healthy B cells.

HS1 nanoclusters were recognized also in the MEC1 CLL line, either by immunofluorescence labeling of the endogenous protein or by overexpression of the YFP-HS1 fluorescent variant, demonstrating that the peculiar HS1 clustering is not antibody-induced or dependent on the overexpression of a YFP-tagged form. At the same time, these parallel experiments also validated the MEC1 line as a suitable model for genetic induction of specific HS1 forms, which cannot be performed in primary cells. Since the coalescence of HS1 clusters at the basal side of primary cells alluded to the binding of the protein to the cytoskeleton as previously described, we further tested the MEC1 model by evaluating the positive co-localization at the STED resolution and the direct interaction with Vimentin by a phasor-FLIM-FRET analysis, which showed an increased interaction at the basal sides.

In the present work, we describe a pioneer approach in the field of B cell biology that paves the way to improve the functional characterization of the active/inactive forms of HS1, their intracellular distribution, nanoscale colocalization, and direct molecular interactions.

Thereby, we decided to limit the present study to cells adhered on polyornithine dishes only, to overcome the complexity of CLL adhesion processes and intrinsic adhesive capacity variability (ten Hacken et al., 2013). Further analysis with other types of ECM components, stromal cell cocultures, and/or selected antigens will be addressed in the future to explore the effect of specific microenvironments on the possible re-localization of HS1 clustering and to study its phosphorylations known to be activated following BCR stimulation.

Ultimately, the future combination of super-resolution 3D imaging with biochemical analyses might provide a robust and quantitative approach for determining whether different cellular distributions of the protein and its phosphorylated forms are associated with distinctive functions, further exploring the difference between leukemic cells and healthy B cells first in CLL and later in other leukaemias and lymphomas, engaging other cytoskeleton adaptors and modulators.

## Data Availability Statement

The raw data supporting the conclusions of this article will be made available by the authors, without undue reservation.

## Ethics Statement

The studies involving human participants were reviewed and approved by the Ospedale San Raffaele (OSR) ethics committee under the protocol VIVI-CLL entitled: “*In vivo* and *in vitro* characterization on CLL.” The buffy coats study was approved by the Ospedale San Raffaele (OSR) ethics committee under the protocol Leu-Buffy coat entitled: “Characterization of leukocyte subpopulations from buffy coats.” The patients/participants provided their written informed consent to participate in this study.

## Author Contributions

MS performed the STED experiments and image analysis. AD did the confocal microscopy and helped in image analysis. MZ developed the IMARIS analysis method and did the FLIM experiments with the analysis. VLC contributed to the STED colocalization experiments. FB prepared the samples. LS provided the patients’ cells and clinical information. CS provided all the reagents and cells and contributed to the experimental plan. VRC supervised the experiments and wrote the manuscript. CS, MZ, and MS revised the manuscript. All authors contributed to the article and approved the submitted version.

## Conflict of Interest

The authors declare that the research was conducted in the absence of any commercial or financial relationships that could be construed as a potential conflict of interest.
