# Supplementary material for: 3D-STED Super-Resolution Microscopy Reveals Distinct Nanoscale Organization of the Hematopoietic Cell-Specific Lyn Substrate-1 (HS1) in Normal and Leukemic B Cells
Source: Front Cell Dev Biol. 2021 Jun 30;9:655773. doi: 10.3389/fcell.2021.655773 (PMC8278786; doi:10.3389/fcell.2021.655773)
Supplement: Supplementary file 5 [file Data_Sheet_1.pdf]

# Supplemental Figures

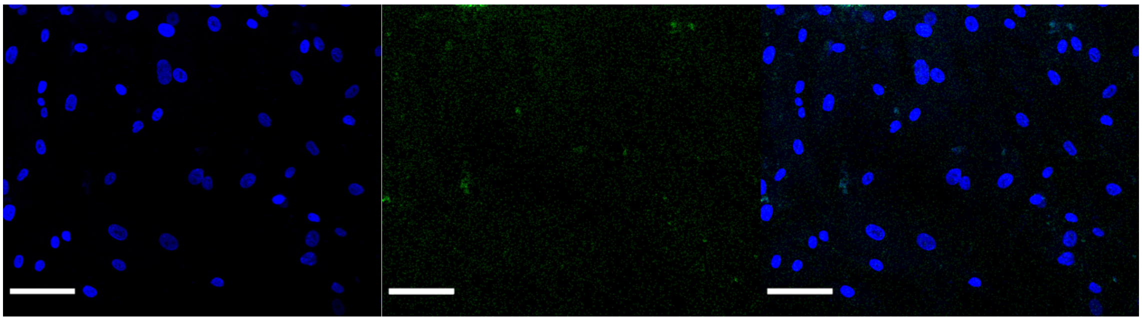


**Figure S1 – HS1 Immunostaining on fibroblasts.**
The specificity of the primary anti-HS1 Ab used for immunolabelling CLL, B and MEC1 cells was tested on fibroblasts expressing high levels of HS1 homologous Cortactin.
Scale bar:100 μm

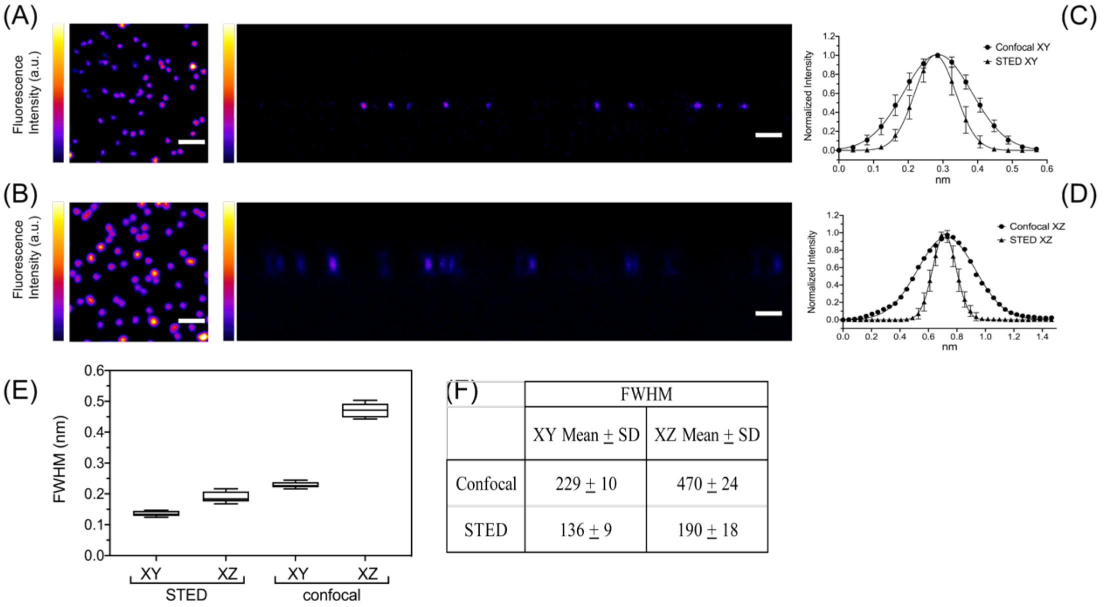


| | FWHM | |
|---|---|---|
| | XY Mean ± SD | XZ Mean ± SD |
| Confocal | 229 ± 10 | 470 ± 24 |
| STED | 136 ± 9 | 190 ± 18 |

**Figure S2 - 3D-STED resolution**
Nanobeads-Alexa488 of 20 nm were used to measure the physical resolution attainable under our working condition in 3D-STED (A) and 3D-confocal (B) images. XY plane (left panels) and XZ plane (right panels). The average PSF (± SD) in the XY (C) and XZ (D) planes were obtained analyzing a minimum of 10 nanobeads. (E) Histogram plot of the Full Width at Half Maximum (FWHM) values (mean ± SD) and (F) average values for each image plane showing the quasi-isotropic PSF (nm) obtained by the applied protocol that resolves the axial dispersion in the 3D-STED stacks. Scale bars 1 μm.

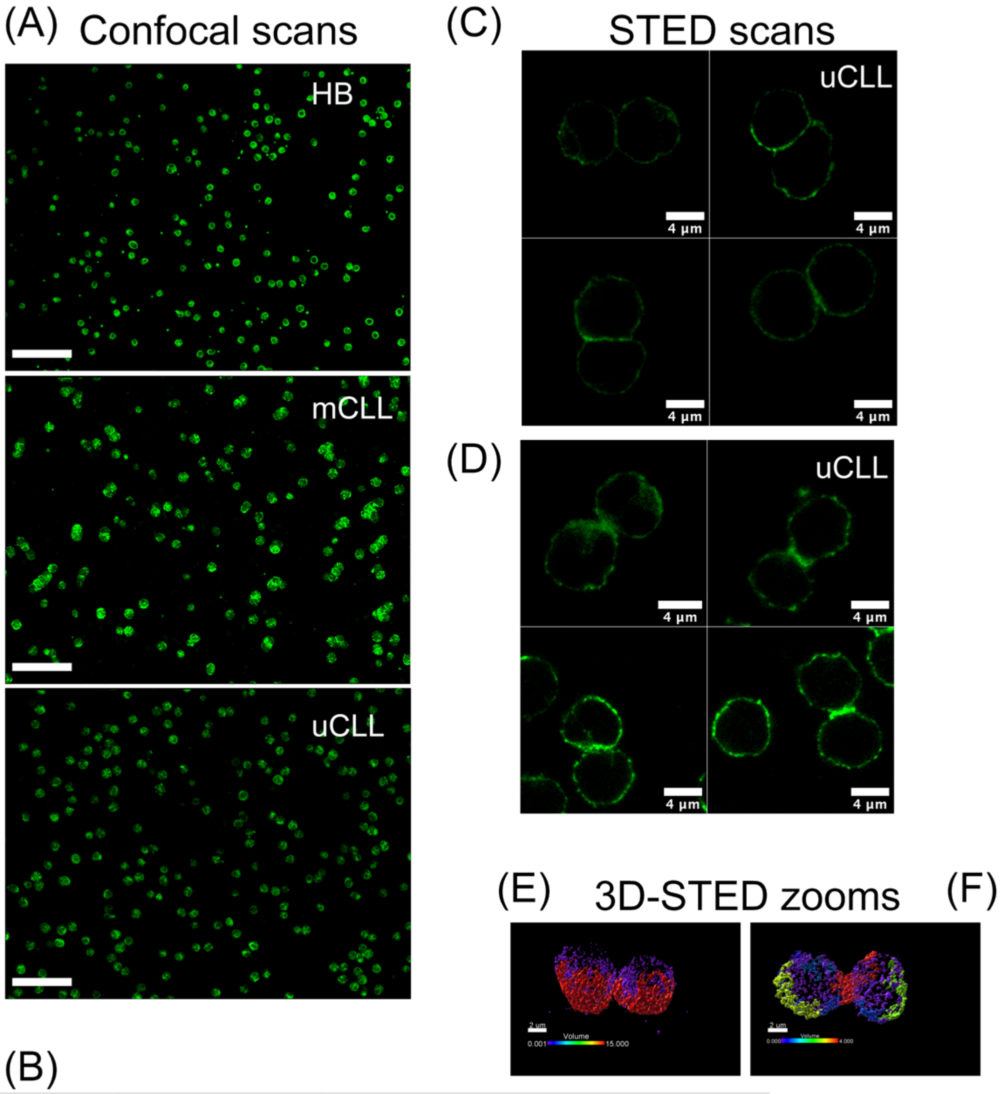


(B)

| | Total Cells | % Cell Contacts | % Synapses |
|---|---|---|---|
| HB | 567 | 0 | 1 |
| mCLL | 499 | 15 | 1 |
| uCLL | 628 | 3 | 1 |

**Figure S3 - Samples subjected to the 3D-STED analysis**

(A) Each cell sample prepared for the 3D-STED was first inspected by confocal microscopy to evaluate cell, integrity, dispersion, density, and staining. Scale bar 50 µm. (B) Cell number and cell pairs of the representative examples shown in (A). By 2D STED, we recognized cell-cell contacts (C) and rarely, immune-synapse like contacts (D), possibly due to a minimum T-cell contamination of the samples. These cells were also examined by 3D-STED, and revealed a clear difference of HS1 gradients. In cell contacts (E), HS1 showed the typical gradient towards the adhesion sites. In contrast, in immune synapse-like cases (F), the highest accumulation of HS1 was observed at the synapse. Although in both cases cells were excluded from the single cell analysis, 3D-STED super-resolution clearly allows distinguishing and characterizing the two events. The volume pseudo color scale is in µm$^3$.

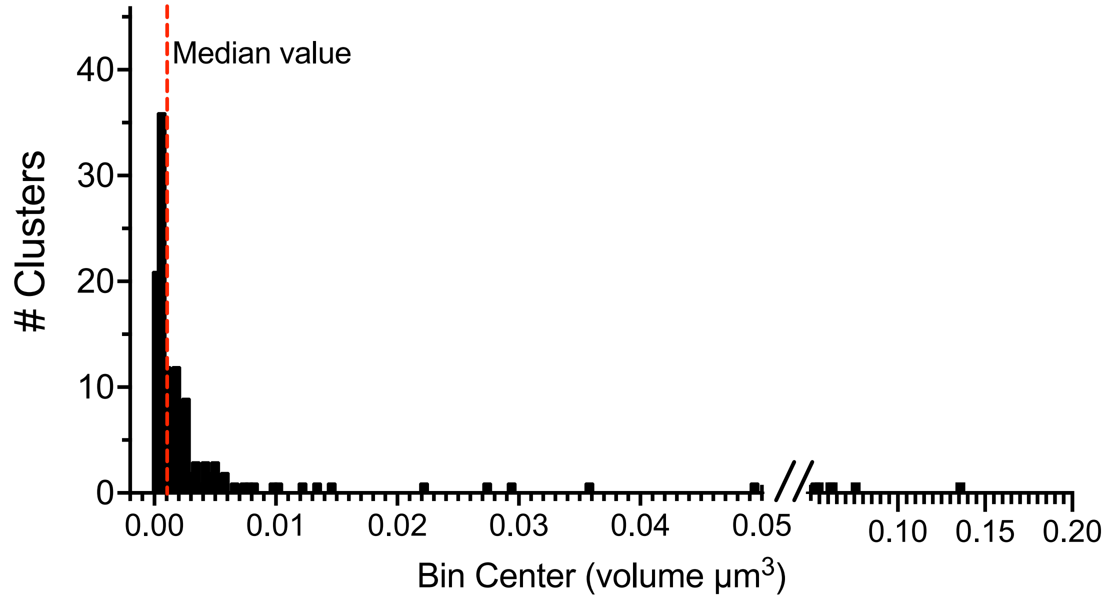


**Figure S4 – Representative example of a cluster size distribution in a single cell central section.**
Histogram of the volume distribution of clusters measured in the central area of the cell #2 from patient P6 (see Supplemental Table S1 for patients' details).

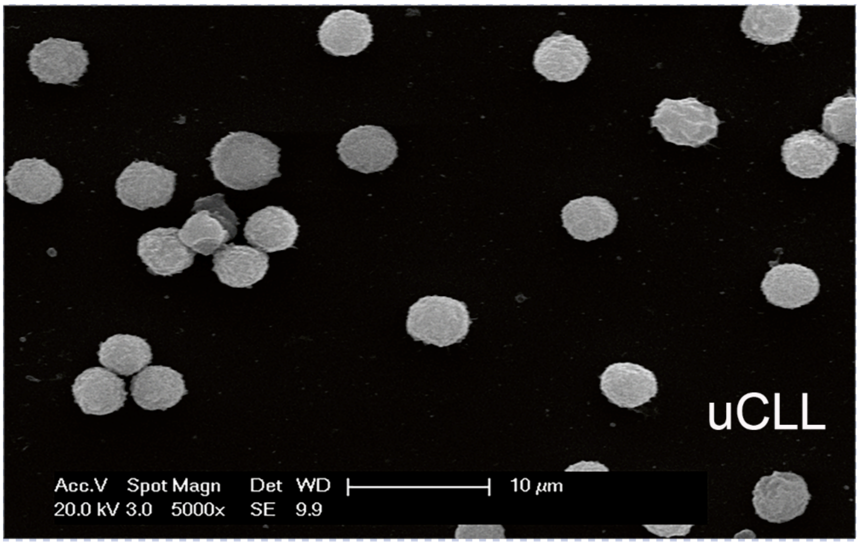


**Figure S5– Visualization of CLL cells by Scanning Electron Microscopy (SEM)**
SEM analysis was performed on uCLL cells. Cells were fixed in 4% paraformaldehyde and 2% glutaraldehyde in phosphate buffer 0.12M, post-fixed in 1% OsO4 in cacodylate buffer, dehydrated in ethanol and then in hexamethyldisilazane. Air-dried samples were gold coated by Edwards S150A (BOC Edwards, Crawley, United Kingdom) and imaged on FEI/Philips XL-30 scanning electron microscope (F.E.I. Company, Hillsboro, OR, USA. [made in Eindhoven, The Netherlands]).

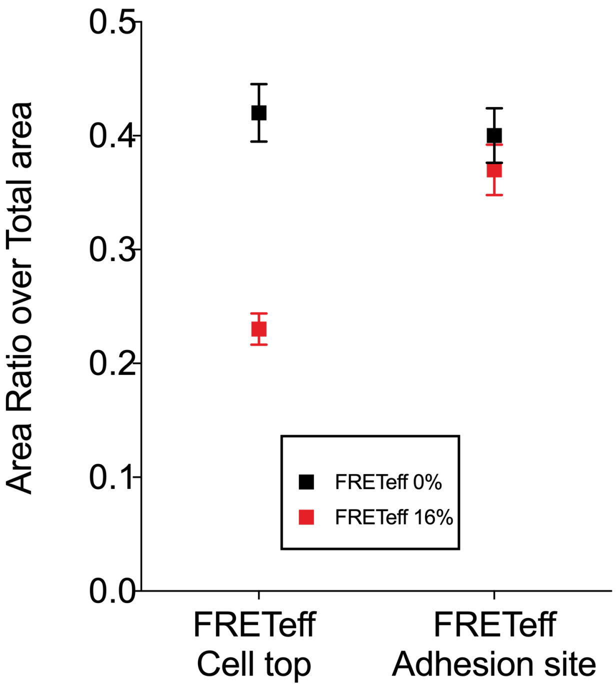


**Figure S6– FRETeff ratios on the representative single cell analysis in Figure 4**
The areas delimited by the phasor plot selection at FRETeff 0 $\pm$6% and 16 $\pm$6% were identified by masks, measured in microns and represented as ratio over the entire optical section. Areas at intermediate FRETeff are not shown.

# Supplemental Tables

## Table S1 – Summary of STED methods.

| Method | | STED Z-stacks central and adhesion sections | Whole Cell STED Z-stacks | | | STED colocalization analysis |
|---|---|---|---|---|---|---|
| ACQUISITION | Reference figures | 1A, 2, 3B, 3C | 3A, 3E | 1D | 4B | 4A |
| | Type of sample | Primary CCL and B cells | Primary CCL and B cells | MEC1 cells | MEC1 cells | MEC1 cells |
| | Sample preparation | Fixed immunlabelled | Fixed immunlabelled | Fixed | Fixed immunlabelled | Fixed immunlabelled |
| | Target | endogenous HS1 | endogenous HS1 | YFP-HS1 | endogenous HS1 and Vimentin | endogenous HS1 and Vimentin |
| | Dye/s | Alexa488 | Alexa488 | YFP | Alexa568 + Alexa532 | Alexa568 + Alexa532 |
| | Depletion mode | X,Y,Z | X,Y,Z | X,Y,Z | X,Y,Z | X,Y |
| | Depletion laser (nm) | 592 | 592 | 592 | 660 | 660 |
| | Voxel size X,Y,Z (nm) | 60 x 60 x 100 | 56 x 56 x 80 | 54 x 54 x 80 | 47 x 47 x 80 | 18 x 18 |
| | Scan mode | XYZ | XYZ | XYZ | XYZ | XY |
| | z stack depth (µm) | 1.0 - 1.30 | 5-7 | 7-8 | 7-8 | - |
| ANALYSIS | Deconvolution (Huygens) | NO | GMLE | GMLE | GMLE | CLME |
| | Exponential fit correction by FiJi (applied only to bleaching < 10%) | YES | YES | YES | YES | NO |
| | Other image post-processing (FiJi) | NO | NO | NO | background | background - Gauss filter 0.5- Coloc 2 |
| | Correction methos - local background subtraction (IMARIS) | YES | YES | YES | YES | - |
| | Correction methos - size cluster filter (IMARIS) | YES | - | - | - | - |

**Table S2 -** IGHV identity of the patients donating CLL cells for the experiments presented in the manuscript (n=11)

| P-identifier | | Patient n° | Provenience | Sample | IGHV identity | Prognosis |
|---|---|---|---|---|---|---|
| P3 | mCLL | 138 | OSR | PB | 88,90 | good |
| P1 | mCLL | 28 | OSR | PB | 89,24 | good |
| P8 | mCLL | 509 | OSR | PB | 91,38 | good |
| P9 | mCLL | 523 | OSR | PB | 92,28 | good |
| P2 | mCLL | 127 | OSR | PB | 92,36 | good |
| P10 | mCLL | 575 | OSR | PB | 97,91 | good |
| P4 | uCLL | 359 | OSR | PB | 99,70 | poor |
| P6 | uCLL | 395 | OSR | PB | 99,70 | poor |
| P5 | uCLL | 376 | OSR | PB | 100,00 | poor |
| P7 | uCLL | 499 | OSR | PB | 100,00 | poor |
| P11 | uCLL | 623 | OSR | PB | 100,00 | poor |

PB: Peripheral blood; OSR: Ospedale San Raffaele, Milan Italy

Patients with CLL were diagnosed according to the updated National Cancer Institute Working Group (NCIWG) guidelines [1] Peripheral blood (PB) samples were obtained after informed consent from patients who were either untreated or off treatment for at least 6 months. The study was approved by the Ospedale San Raffaele (OSR) ethics committee under the protocol VIVI-CLL entitled: “In vivo and in vitro characterization on CLL”. The buffy coats study was approved by the Ospedale San Raffaele (OSR) ethics committee under the protocol Leu-Buffy coat entitled: “Characterization of leukocyte subpopulations from buffy coats”. Clinical and biological characteristics of patients with CLL who provided samples for the experiments are reported in **Table S2**.

**Supplemental Reference**

[1] M. Hallek, B.D. Cheson, D. Catovsky, F. Caligaris-Cappio, G. Dighiero, H. Dohner, P. Hillmen, M.J. Keating, E. Montserrat, K.R. Rai, T.J. Kipps, and L. International Workshop on Chronic Lymphocytic, Guidelines for the diagnosis and treatment of chronic lymphocytic leukemia: a report from the International Workshop on Chronic Lymphocytic Leukemia updating the National Cancer Institute-Working Group 1996 guidelines. Blood 111 (2008) 5446-56.

## Supplemental Movies

**Movie S1**

3D rendering of Alexa488-HS1 in a whole B cell Z-stack acquired by forward and backward scanning.

**Movie S2-S3-S4**

3D-STED stacks representing immunolabelled Alexa488-HS1 samples of mCLL cells (patient P3), uCLL cells (patient P4), and healthy B cells (donor H5). Scale bar 10 μm. (see Supplemental **Table S2** for patients' details).

## Supplemental Methods

**Confocal Microscopy**

We used a gated WLL SP8-LasX-Navigator laser scanning microscope equipped with a HC PL APO CS2 63x/1.40 oil objective and a HyD spectral detector in standard mode set to collect the emission 504-546 nm window. Excitation was at 498 nm. Tiled X,Y,Z scanned volumes of 340.43x250.96x1.4 µm were acquired in 15 optical sections with a voxel size of 45x45x100 nm at 400 Hz unidirectional mode, and zoom 2. Results are shown as average projections.
